# PTT-quant: a new method for direct identification and absolute quantification of premature transcription termination events, following the example of bacterial riboswitches

**DOI:** 10.1007/s00253-022-11809-1

**Published:** 2022-02-05

**Authors:** Piotr Machtel, Anna Wasilewska-Burczyk, Julian Zacharjasz, Kamilla Grzywacz

**Affiliations:** Institute of Bioorganic Chemistry Polish Academy of Sciences, Poznań, Poland

**Keywords:** Transcription termination, Riboswitch, *Bacillus subtilis*, Transcription regulation

## Abstract

Regulation of gene expression by premature termination of transcription has been well described in all domains of life, including metazoans, yeast, plants, and bacteria. Although methods for identification of such regulatory events by sequencing are available, the focused biochemical studies of the mechanism are hampered by lack of highly sensitive and accurate experimental methods. Here, we propose a new method for absolute quantification of premature transcription termination events, PTT-quant. It is based on highly sensitive two-step digital droplet PCR protocol, coupled with normalized cDNA synthesis attained by site-specific pre-cleavage of investigated transcripts with RNase H. As a consequence, our method enables the reliable and sensitive quantification of both, prematurely terminated and full-length transcripts. By application of our method to investigation of transcriptional riboswitches in *Bacillus subtilis*, we were able to precisely measure the dynamics of S-adenosylmethionine (SAM) riboswitch induction, which turned to be ~ 23% higher in comparison the results obtained without cDNA synthesis normalization.

**Key points**

• A novel method for quantification of premature transcription termination events was established.

• PTT-quant measures absolute concentration of full-length and terminated transcripts.

• RNase H and the digital droplet PCR technique is used in PTT-quant.

**Supplementary Information:**

The online version contains supplementary material available at 10.1007/s00253-022-11809-1.

## Introduction

The premature transcription termination (PTT) has been discovered decades ago, but only recently, it has been recognized as one of the major regulatory mechanism, leading to the diversification of the gene expression. Up to date, the PTT has been reported in all kingdoms of life. In *Eukaryotes*, it can lead to the synthesis of both, stable and unstable products. Depending on the gene region where the termination occurs, two different types of the PTT can be distinguished. The transcription start site (TSS)-linked PTT is most likely related to the low efficiency of the full-length gene transcription by the polymerase II (Pol II), resulting in high turnover within TSS regions (Krebs et al. 2017; Steurer et al. 2018). This phenomenon leads to the synthesis of a substantial amount of short, capped, mostly unstable TSS-proximal RNAs of yet unknown function, which in a way similar to promoter upstream transcripts (PROMPT) contribute to the exosome-sensitive RNAs. In contrast, premature transcription termination occurring within the coding sequence (CDS) region is mostly related to the occurrence of the cryptic alternative polyadenylation (APA) sites. Among those, we can distinguish the coding sequence polyadenylation (CDS-APA) and the intronic polyadenylation (IPA) sites. Their employment is widespread, e.g., in mouse ~ 40% of genes are subjected to premature transcription termination within the gene body (Hoque et al. 2013). The PTT events occurring closer to the 5ʹ part of the gene tend to produce noncoding RNAs, whereas later PTTs result rather in a synthesis of the truncated proteins (Singh et al. 2018). Most of such transcripts are capped and polyadenylated, playing important roles in cell metabolism. For example, the PTT has been shown to form mRNA variants encoding soluble isoforms of the transmembrane T-cell co-stimulator CD46 (Ly et al. 2017).

In bacteria, premature transcription termination is mostly related to the activity of the RNA-based cis-regulatory mechanisms. One of the best studied are the riboswitches — highly structured mRNA domains with the unique capability of direct binding of the small metabolites. Such binding event leads to the changes in the mRNA secondary structure and evokes regulatory effect (Lin and Thirumalai 2012). Most riboswitches operate on the principle of negative feedback loops by their placement within the mRNAs encoding for the proteins which are involved in maintaining homeostasis of a recognized metabolite, including its biosynthesis (Sudarsan et al. 2003), transport (Hullo et al. 2004), or secondary metabolism (Mandal et al. 2004). Such regulation utilizes a broad spectrum of mechanisms at various stages of the gene expression, including transcription, translation, mRNA degradation, splicing, or acting as small RNAs (Machtel et al. 2016). Importantly, the ligand:riboswitch interaction can silence (Winkler et al. 2002) or activate gene expression (Tripp et al. 2009). To date, nearly 45 different classes of riboswitches were described (McCown et al. 2017). Although riboswitches are considered as typical for bacteria (Mellin and Cossart 2015; Winkler and Breaker 2005; Pavlova et al. 2019), they have been identified among all domains of life (Gupta and Swati 2019; Wachter 2010; Mukherjee et al. 2018).

Despite the wide wealth of the regulatory mechanisms, the vast majority of currently known riboswitches act via the premature transcription termination (Naville and Gautheret 2010). In the apo (unbound) riboswitch state, the expression platform adopts a conformation with a dominant anti-terminator structure, which allows for an undisturbed process of the mRNA transcription and the production of the full-length mRNAs. Binding of a ligand (holo state) triggers the conformational alteration of a riboswitch, resulting in the formation of a terminator hairpin structure, which in turn leads to the PTT and the synthesis of short (truncated) nonfunctional mRNAs and gene silencing (Garst et al. 2011).

The identification of the premature transcription termination events is in most cases fulfilled with the employment of the new generation sequencing (NGS)-based techniques, being variations of the 3ʹ-directed RNA-seq. The quantification of the PTT efficiency using those methods is however affected by multiple experimental biases introduced during individual steps of cDNA library preparation, including differences in efficiency of 3ʹ adapter ligation, PCR amplification length bias, cDNA library composition, and others. Thus, the identified PTT sites require further verification with the employment of independent methods. Many of the currently employed approaches depend on the quantification of the changes in full-length transcript levels, ignoring the concentration of truncated transcripts (Tomsic et al. 2008). In this way, the information concerning the actual activity of the PTT event is lost. Moreover, measured changes in the transcript levels are the resultant of multiple cellular processes, including the activity of the RNA polymerase or the RNA turnover (Mansilla et al. 2000; Even et al. 2006). Thus, to estimate the efficiency of the PTT event both fractions of the transcripts (that is, full-length and truncated) should be quantified and their ratio should be estimated.

**Fig. 1:**
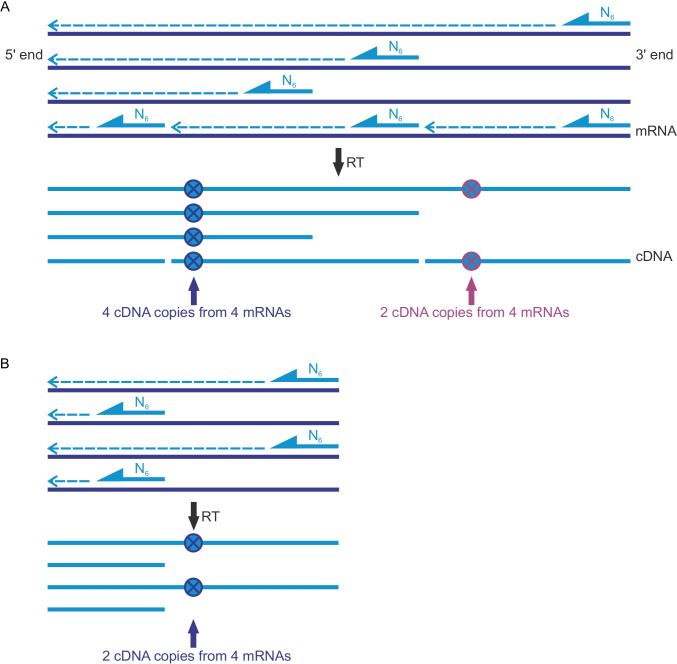
The dependence of the number of the cDNA copies on the distance from the 3ʹ end of the transcript. Random hexamers (N_6_) hybridize to the mRNA molecules in a stochastic manner. The synthesis of the cDNA on the mRNA template occurs in a 3ʹ → 5ʹ direction of the mRNA (dashed arrows), which is the cause of an unequal coverage of the mRNA with cDNA copies. There is a high probability that regions of mRNAs closer to the 3ʹ ends will be converted into cDNA with lower frequency, compared to the 5ʹ ends. As a result, a strong bias exists in a quantification of the number of detected cDNA copies for both full-length transcripts (**A**) and prematurely terminated transcripts (**B**)

When aiming to accurate detection of terminated and full-length transcripts, several issues must be taken into consideration. Routinely exploited hybridization-based techniques, like northern blot hybridization, although enable the amplification-free estimation of the mRNA levels, are hampered by several limitations, e.g., the inability for controlling the efficiency of the RNA transfer from the gel to the membrane, RNA-membrane binding, and differences in the kinetics of the detection probe hybridization to the targeted nucleic acids (Fernyhough 2001; Reue 1998). Moreover, northern blot analysis can cause RNA degradation during the electrophoresis steps. All those issues contribute to the low sensitivity and resolution, much lower in comparison to the amplification-based techniques (Streit et al. 2009). Thus, the most frequently employed technique for the PTT investigation is a reverse transcription followed by a quantitative PCR (RT-qPCR), aimed at detection of both, prematurely truncated and full-length transcripts. Although the RT-qPCR method provides high sensitivity, it is hampered by biases during the cDNA synthesis. In one-step RT-qPCR protocol, the same set of starters are used for the reverse transcription and the PCR reactions. Thus, the comparative analysis of prematurely terminated and full-length transcripts cannot be performed from a single RNA sample. The requirement of using two separate RNA samples, even if obtained from the same RNA pool, could lead to the measurement errors, which influence the estimated PTT efficiency. Alternatively, a two-step RT-qPCR protocol can be applied, where the cDNA is synthetized using random hexamers as starters, and next, on a single cDNA pool, two PCR reactions can be performed. This approach, however, reveals a systematic bias towards the overrepresentation of the 5ʹ ends of transcripts during cDNA synthesis (Agrawal et al. 2015). The reason for this phenomenon is the fact that the reverse transcriptase synthesizes the cDNA in the direction from the 3ʹ to the 5ʹ end (in relation to the transcripts); therefore, there is a high probability that regions of mRNAs closer to the 3ʹ ends will be converted into cDNA with lower frequency, compared to 5ʹ ends (Fig. 1). Thus, the results of the quantification depend not only on the amount of the RNA, but also on the position of the of the PCR primers target sites within the transcript. The above effect introduces bias in a relative quantification of the full-length (Fig. 1A) and prematurely terminated transcripts (Fig. 1B). It specifically concerns the transcripts with a significant length and could contribute to the artificial overestimation of the PTT transcripts.

Due to the above limitations in the currently available protocols, we decided to establish a novel method for the quantification of the premature transcription termination events, which will be flexible, accurate, sensitive and less prone to the introduction of biases. As a model, we have employed S-adenosylmethionine (SAM) binding riboswitches in *Bacillus subtilis*. The bacterial transcriptional riboswitches are well suited for such task, due to the relatively straightforward control over the PTT activity controlled by a ligand concentration. Also, the exact position of the PTT events in the sequence can be easily estimated by the identification of the attenuator hairpin following an investigated riboswitch’s aptamer domain.

## Materials and methods

### Primers and DNA oligomers

Primers for the PCR (Supplemental Table S1) were designed using Primer BLAST tool and *B. subtilis* 168 strain genome sequence as a reference (NCBI database accession number AL009126.3). DNA oligomers used for RNase H cleavage reaction are presented in Supplemental Table S2.

### Bacterial strain and growth conditions

The *B. subtilis* 168 strain was used in the study. For a routine growth, the *B. subtilis* cells were grown on the sporulation medium (SP) or ED minimal medium (Belda et al. 2013). Bacteria were grown at 37 °C to OD_600_ = 0.6. The methionine elimination was performed as follows: *B. subtilis* was grown to OD_600_ = 0.6, and the pellet was centrifuged and resuspended in ED medium lacking methionine. The culture was continued for up to 4 h.

### DNA isolation

The genomic DNA (gDNA) was isolated from 2 ml of *B. subtilis* bacterial culture with OD_600_ from 0.8 to 1. The bacteria were centrifuged, and a pellet was resuspended in 500 μl of cold DNA isolation solution (50 mM glucose, 2.5 mM Tris–HCl pH 8.0, 10 mM EDTA pH 8.0, 2 mg/ml lysozyme, 100 U RNase A, Thermo Fisher Scientific, Waltham, USA) and incubated for 10 min on ice. Then 50 µl of 10% SDS was added, and the resulting mixture was incubated at 37 °C for 10 min. gDNA was phenol/chloroform extracted. DNA quality and quantity were verified with NanoDrop spectrophotometer (Thermo Fisher Scientific, Waltham, USA) and gel electrophoresis.

### RNA isolation

RNA was isolated with GeneMATRIX Universal RNA purification kit (EURx, Gdansk, Poland), according to the manufacturer’s protocol. The RNA quality and quantity were measured with NanoDrop spectrophotometer and verified with gel electrophoresis.

### PCR

PCR templates for an in vitro transcription and ex vivo RNase H cleavage were amplified using a DreamTaq PCR Master Mix 2x (Thermo Fisher Scientific, Waltham, USA). One microgram of *B. subtilis* genomic DNA was used as a template, and the final concentration of the primers equaled 200 pM each. The PCR products were purified with GeneJET PCR Purification Kit (Thermo Fisher Scientific, Waltham, USA), and their purity was verified on 1% agarose gel with SYBR Safe reagent (Thermo Fisher Scientific, Waltham, USA) and visualized on G-Box Chemi XR5 system (Syngene Bangalore, India).

### In vitro* transcription*

In vitro transcription of the PCR products was performed with MEGAscript™ T7 kit (Thermo Fisher Scientific, Waltham, USA) using a 45 nt-long primer (5ʹ-TAATACGACTCACTATAGGGAGAATGAGTGAACAAAACACACCAC-3ʹ). Eight microliters of the PCR template was used; the final concentration of NTPs equaled 7.5 mM each. The samples were incubated for 4 h at 37 °C. Subsequently, in vitro transcription products were purified with GeneMATRIX Universal RNA purification kit (EURx, Gdansk, Poland) and visualized on a 10% polyacrylamide (PAA) gel with SYBR Safe reagent (Thermo Fisher Scientific, Waltham, USA).

### RT-PCR

RT-PCR was conducted using SuperScript IV Reverse Transcriptase (Thermo Fisher Scientific, Waltham, USA). The RNA molecules obtained ex vivo were used as templates. The synthesis of the cDNA was carried out with the use of 2.5 μM random hexamer primers, 0.5 mM dNTP each, and 1 ng of the RNA template. The reaction mix was incubated at 65 °C for 5 min and on ice for 1 min. Subsequently, 4 µl of SuperScript IV RT buffer, 1 µl of 5 mM DTT, 0.5 µl of RNaseOUT Recombinant RNase Inhibitor (40 U/µl) (Thermo Fisher Scientific, Waltham, USA), and SuperScript IV Reverse transcriptase (200 U/µl) (Thermo Fisher Scientific, Waltham, USA) were added. The samples were incubated in a thermocycler at 23 °C for 10 min, 50–55 °C for 10 min, and at 80 °C for 10 min.

### RNase H cleavage

DNA oligomers complementary to the selected RNAs were designed using Primer BLAST tool (Supplemental Table S2). One μg of the transcript template was incubated with 100 pM to 5 µM DNA oligomers (depending on the experiment) for 20 min at 37 °C. The hydrolysis of the DNA-RNA duplex was performed using 1.25 U Ribonuclease H (Thermo Fisher Scientific, Waltham, USA).

### cDNA synthesis

For the cDNA synthesis, SuperScript IV Reverse Transcriptase (Thermo Fisher Scientific, Waltham, USA) transcription kit was used. The reaction was carried out according to the manufacturer’s protocol with the use of 50 µM random hexamers. To each experiment, 100 ng of the RNA was used as a template. The first part of reaction, containing the RNA template, random hexamers, and dNTPs, was incubated at 65 °C for 5 min and put on ice, followed by incubation at 23 °C for 10 min, 55 °C for 10 min, and 80 °C for 10 min with remaining reaction components.

#### Droplet igital PCR

Droplet digital PCR (ddPCR) reaction was performed as previously described (Mleczko et al. 2019) with some modifications. ddPCR mix contained: 10 µl of QX200^™^ ddPCR^™^ EvaGreen Supermix (Bio-Rad, Hercules, USA), 4 µM of forward and reverse primers, and 1 µl of cDNA in 20 µl total volume. The reaction mixture was emulsified into droplets using a QX100 Droplet Generator (Bio-Rad, Hercules, USA) according to the manufacturer’s protocol. PCR was performed according to the program: 5 min at 95 °C (followed by 40 cycles of 30 s at 95 °C, 30 s at 55 °C, and 45 s at 72 °C) and then 2 min at 72 °C, 5 min at 4 °C, and 5 min at 90 °C and finally kept at 12 °C. The samples were analyzed using the QX100 Droplet Reader (Bio-Rad, Hercules, USA) and QuantaSoft software (Bio-Rad, Hercules, USA).

#### RT-qPCR

RT-qPCR was performed as previously described (Bakowska-Zywicka et al. 2016) with some modifications. RT-qPCR mix contained: 4 µl of 5 × HOT FIREPol EvaGreen qPCR Mix Plus (Solis Biodyne, Tartu, Estonia), 200 nM of forward and reverse primer each, and 5 µl of cDNA samples (dilution 1:50). The datasets were collected on Applied Biosystems QuantStudio 6 Flex Real-Time PCR System (Thermo Fisher Scientific, Waltham, USA). The cycling conditions were as follows: 12 min at 95 °C, followed by 40 cycles consisting of 15 s at 95 °C, 20 s at 60 °C, and 20 s at 72 °C. The fluorescence signal data were collected during the 72 °C phase of each cycle. The specificity of the amplified targets was assessed by melting curve analysis from 55 to 95 °C (in 0.5 °C increments, measuring fluorescence at each temperature) following the last cycle. The analysis showed the presence of only one specific product in each reaction. All primer pairs were tested with regard to the amplification efficiency with 4 × log10 serial dilution of a random cDNA sample in triplicates. All tested primers met the criteria of efficiency 90–110% and *R*^2^ > 0.985. The results were expressed as a relative quantity according to the equation RQ = 2^ΔΔCt^.

## Results

### Protocol design

Among high sensitivity amplification-based techniques, the reverse-transcriptase digital droplet PCR (RT-ddPCR) seems to be the best suited for the investigation of the PTT activity, due to the superior accuracy, without the need for the standard curve estimation, obtained by an absolute quantification of the nucleic acids. It also enables the employment of the two-step protocol, consisting of an initial reverse-transcription of the total RNA with the employment of random hexamers, followed by the multiple target-specific ddPCR-based cDNA quantifications from a single cDNA sample. This method is however biased by an overrepresentation of the 5ʹ RNA regions, as a result of non-uniform cDNA synthesis, similarly to the classical two-step RT-qPCR (Fig. 1). The severity of this bias observed in RT-ddPCR results has been shown by Abachin et al. (2018). During the optimization of the RT-ddPCR workflow for the detection of dengue virus, they investigated the efficiency of the RT-ddPCR with the employment of the probes designed toward different regions of the viral RNA genome. The results demonstrated a clear drop in assay sensitivity for assays based on probes located closer to 3ʹ end, especially within 3ʹ UTR.

**Fig. 2:**
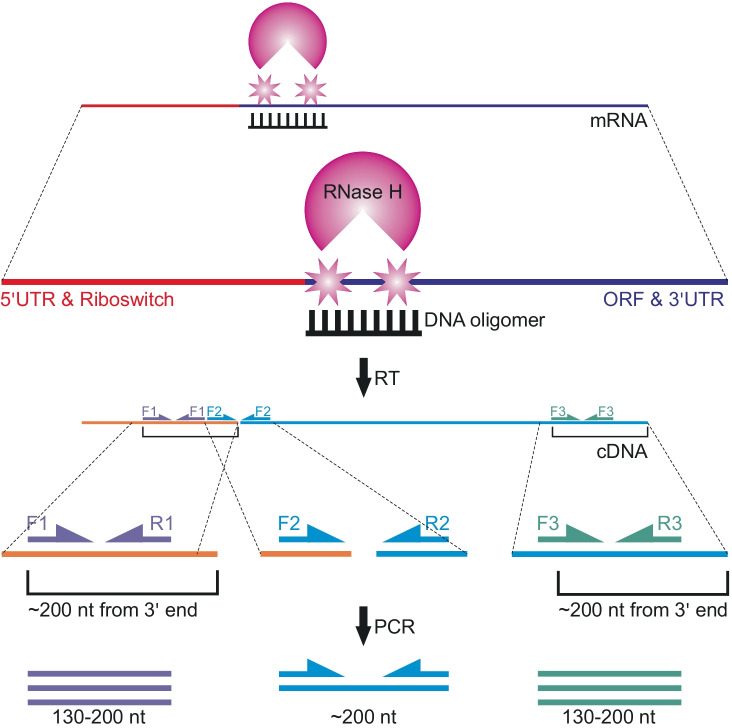
Schematic representation of a new method for the analysis of the premature transcription termination transcriptional activity and primers/oligomer design. mRNA (violet/red line) is cleaved by RNase H (pink circle) downstream the riboswitch (red line), in a position indicated by a DNA oligomer. Transcript levels are measured in a ddPCR reaction, preceded with the reverse transcription. First pair of primers (F1/R1) is complementary to the region upstream of the riboswitch sequence (5′ UTR and riboswitch), and therefore, it allows for the amplification of the full-length and terminated transcripts. The second pair of primers (F2/R2) flanks the RNase H cleavage site and serves as a control of the RNase H cleavage efficiency. The third pair of primers (F3/R3) is complementary to the region downstream the riboswitch sequence (ORF and 3′ UTR), and it allows for amplification of the full-length transcripts solely

In order to eliminate the influence of the 3ʹ end distance on the number of the reads (Fig. 1), we decided to use an endonucleolytic cleavage of the investigated full-length transcript with RNase H at the position defined by designed DNA oligomers, complementary to the sequences near the natural PTT site (Fig. 2). As the result, we obtained two types of RNAs: one representing the 5ʹ part of the transcript and second representing the 3ʹ part, being an extension beyond the investigated PTT site. Thus, the quantification of the first transcript provides an information about the total expression of the gene, whereas quantification of the second transcript provides information about the full-length transcription. By designing the ddPCR probes for the detection of both transcript isoforms at the positions in the same distance from their 3ʹ ends, we were able to ensure an unbiased efficiency of the cDNA synthesis for both probe sets, independently from the measured transcript length.

In our experiments, we have designed 3 pairs of primers for each investigated transcript. The first pair of primers (Fig. 2, primers F1 and R1) is complementary to the riboswitch aptamer sequence at 5′ untranslated region of the mRNA (5ʹ UTR), upstream of the PTT site. The second pair of the primers (primers F2 and R2) flanks the transcription termination site, whereas the third pair (primers F3 and R3) was designed to bind to the open reading frame (ORF) of the mRNA sequence. Primers F1/R1 and primers F3/R3 were designed to hybridize at the same distance (~ 200 nt, Fig. 2) from the 3ʹ end of the RNase H cleavage product and full length transcript, respectively. In this way, the reads from primers F1/R1 can be used for the quantification of the sum of the full-length and terminated transcripts, primers F3/R3 provide the information about the amount of the full-length transcripts solely, whereas primers F2/R2 provide the control for the RNase H cleavage reaction. The difference between the number of the reads from the first and the third pair of primers gives the information on the number of the truncated transcripts, which are produced as a result of the riboswitch-induced PTT activity.

### *Optimization of the efficiency and the specificity of the RNase H cleavage of *in vitro* transcripts*

To optimize the efficiency and the specificity of the RNase H cleavage, we prepared a set of the cleavage reactions in vitro. To directly observe the action of the RNase H, in the first step, we synthesized model transcripts with the use of an in vitro transcription reaction on a PCR-amplified templates containing the T7 promoter for RNA polymerase, coupled with a partial sequence of the *gyrA* gene composed of 5 ʹUTR and a fragment of a downstream ORF sequence. The RNase H cleavage site was defined by the design of DNA oligomers, selectively complementary to the target RNA sequence near the native PTT site caused by the riboswitch activity (i.e., behind the terminator hairpin and poly-U tract).

Three sizes of DNA oligomers were tested (15 nt, 20 nt, 25 nt, Supplemental Table S2) to determine the impact of the length of the DNA oligomers on RNase H cleavage efficiency (Supplemental Fig. S1). The analysis showed no significant differences in the cleavage efficiency. The only visible differences concerned the size of the analyzed products — as expected from the RNase H cleavage. The reason is the fact that RNase H induces more than one nucleolytic cleavage site, along the whole RNA:DNA heteroduplex. Therefore, oligonucleotides of ~ 20 nt in length were used for further analysis.

In the next step, the concentration of the DNA oligomers was optimized, so that the RNAse H cleavage reaction proceeded in nearly 100% efficiency. The results indicate that most efficient cleavage by RNase H occurs at DNA oligomer concentration of 100 nM (Supplemental Fig. S2, lane 7). Thus, the optimal DNA:RNA molar ratio was estimated as 1:10. Further increase of the DNA oligomer concentration caused a gradual degradation of the cleavage product and an appearance of a shorter product, probably due to the unspecific binding of the oligomer induced by its high concentration.

### Ex vivo* transcripts are efficiently cleaved by RNase H*

Previous experiments proved that the designed DNA oligomers are able to efficiently and precisely determine the RNase H site of in vitro synthesized transcripts. However, the actual purpose of the proposed method was the analysis of the natural transcripts in the pool of the total RNA extracted from the cells (called here ex vivo transcripts). To provide a sufficient sensitivity for the visualization of the results, we have employed the RT-PCR reaction using three pairs of PCR primers targeted towards 5ʹ and 3ʹ parts, as well as the cleavage site (as shown on Fig. 2) of the control *gyrA* transcript in the pool of ex vivo transcripts. In the first step, a range of DNA oligomer concentrations was tested, from 10 nM to 5 µM (Supplemental Fig. S3). The observable amount of the PCR product amplified by primers 2 (F2/R2, flanking the cleavage site) were significantly reduced, starting from the DNA oligomer concentration of 0.1 µM. It suggests the efficient cleavage of a control *gyrA* transcript in the pool of ex vivo transcripts by RNase H. Simultaneously, a general lack of the changes in the intensity of the signals derived from the products amplified by primers 1 and 3 (up- and downstream of the cleavage site, respectively) indicated that the RNase H cleavage of a *gyrA* transcript in the pool of ex vivo transcripts was site-specific. The PCR product amplified by primers 1 and 3 showed a gradual decrease as the concentration of DNA oligomer increases, starting from a concentration of 500 nM, until a complete disappearance at 5 µM. In the lanes with the highest DNA oligomer concentrations, a band signal disappeared, probably due to the high concentration of DNA oligomer, which might interfere with cDNA synthesis or PCR amplification.

**Fig. 3:**
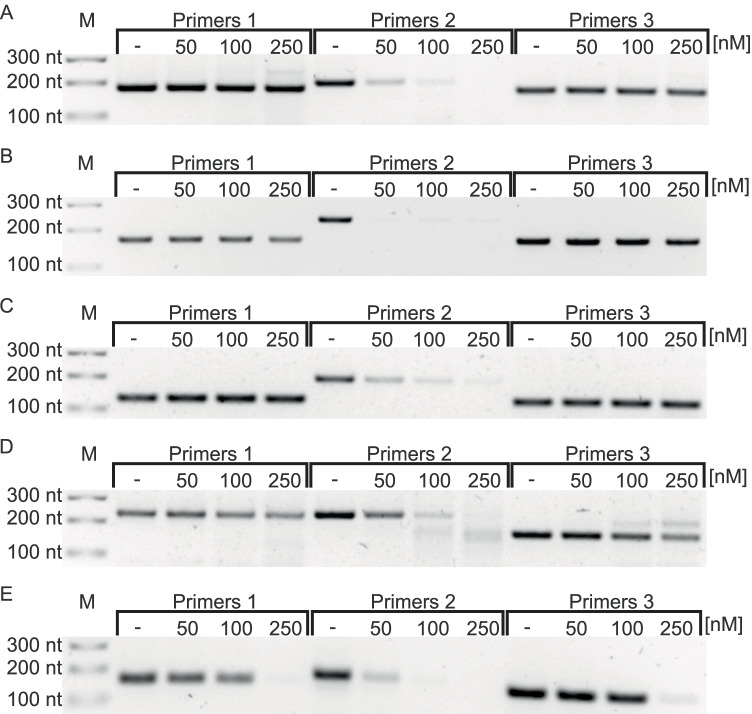
SAM riboswitch-containing transcripts are efficiently cleaved by RNase H ex vivo. RNase H cleavage of SAM riboswitch-containing transcripts in the pool of ex vivo transcripts was performed in a gradient of DNA oligomer concentrations (50–250 nM), for each of the analyzed genes: *gyrA* (**A**), *samT* (**B**), *metIC* (**C**), *metE* (**D**), and *mtnKA* (**E**). A clear disappearance of the signals from products amplified solely by primers 2 (F2/R2) is observed, which indicates specific RNaseH cleavage. The signals derived from the PCR products of primers 1 and 3 are not significantly decreased. M, DNA size marker

Based on the above results, for further experiments, we have chosen a range of DNA oligomer concentrations from 50 to 250 nM, that is for which the changes in the cleavage efficiency were observed for *gyrA* transcript in the pool of ex vivo transcripts. In the next step, we have compared the influence of the DNA oligomer concentration on the cleavage of the SAM riboswitch-containing mRNAs: *samT*, *metIC*, *metE*, and *mtnKA*. For all tested transcripts, the optimal concentration was in a range of 50–100 nM, similar to the *gyrA* transcript (Fig. 3A–E). We observed a disappearance of the signals from products amplified by primers 2 (F2/R2) already after an addition of the lowest DNA oligomer concentration (50 nM). The signals derived from the PCR products of primers 1 and 3 were not significantly decreased even with increasing DNA oligomer concentration, except of *metE* (Fig. 3D) and *mtnKA* (Fig. 3E) transcripts, where a reduced signal was observed only with 250 nM DNA oligomers.

We concluded that the most optimal concentration of the DNA oligomer depends slightly on the gene/operon and equals to 50 or 100 nM per 100 ng of the total RNA. These are the conditions for which a significant reduction (or a disappearance) of the amplification signal of primers 2 and the permanence of signals from primers 1 and 3 are simultaneously observed.

### *ddPCR allows for a quantitative analysis of the RNase H cleavage of *ex vivo* transcripts*

Because the analysis of the PCR products has, at best, a semiquantitative character, we introduced a quantitative ddPCR analysis to the proposed protocol.

We performed RNase H cleavage reactions of *gyrA*, *samT*, *metE*, *metIC, and mtnKA* transcripts in the pool of ex vivo transcripts followed by ddPCR (Supplemental Fig. S4). We observed a visible reduction in primers 2 amplification product concentrations in all tested transcripts already after adding the smallest amount of the DNA oligomer (50 nM). Moreover, when applying ddPCR for the quantification of the 5ʹ and 3ʹ parts of the cleaved transcripts (amplified with the primer sets 1 and 3, respectively), we did not observe any decrease of the products with an increasing DNA oligomer concentration, as it was in the classical RT-PCR. This observation strongly supports the superiority of ddPCR-based quantification over classical RT-PCR method. Furthermore, the employment of the ddPCR allowed for a precise quantification and calculation of the RNase cleavage efficiency. The average cleavage efficiency oscillated around 83%, 97%, and 97% for *gyrA* control transcript and 94%, 96%, and 95% for riboswitch-contained transcripts (for DNA oligomer concentrations of 50 nM, 100 nM, and 250 nM, respectively).

### RNaseH/ddPCR method allows for determination of SAM riboswitches induction under methionine starvation

**Fig. 4:**
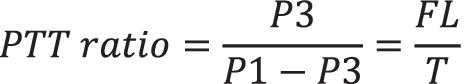
The PTT ratio calculation. FL — concentration of full-length transcript. T — concentration of PTT transcripts. P1, P3 — the concentration of the products amplified with the primers 1 and 3, respectively

The analysis of the RNase H cleavage efficiency by ddPCR revealed that 50 or 100 nM of DNA oligomers per 100 ng of total RNA is the optimal amount, as a significant reduction in the concentration of amplification products from primers 2, whereas the permanence of the signal from primers 1 and 3 was retained. We have therefore decided to employ our newly established method to study the dynamics of SAM riboswitch activity in *B. subtilis*, as a response to the progressing methionine starvation. We used 4 different time-points of methionine elimination from bacterial culture; extracted the total RNA; performed the RNase H cleavage reactions targeted against ex vivo *gyrA* (non-riboswitch control), *samT*, *metIC*, *metE*, and *mtnKA* transcripts; and calculated an absolute quantities of the resulting ddPCR amplification products. The concentration of the prematurely terminated transcripts was calculated as a difference between the concentration of the ddPCR product amplified with the primers 1 and 3. The ratio of the concentrations between the full-length and prematurely terminated transcripts served as a PTT ratio (Fig. 4).

**Fig. 5:**
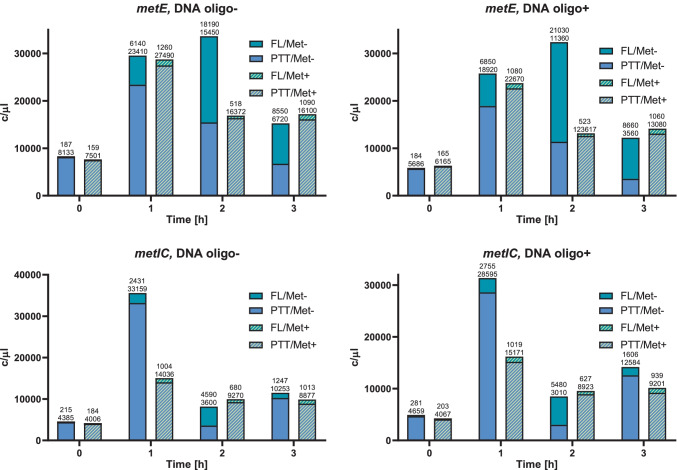
RNaseH/ddPCR method allows for determination of SAM riboswitches induction under methionine starvation. The concentration of the riboswitch-containing transcripts at different time points of methionine starvation is expressed in copies of RNA per µl [c/µl]. The graphs show changing proportion between the full-length (FL) and prematurely terminated (PTT) transcripts as a response to the methionine starvation at four time points (0 h to 3 h)

The analysis of the concentration of individual transcripts, expressed in copies/µl, proved that in vast majority of samples, the concentration of total number of transcripts (the sum of the full length and the truncated) was comparable, with minor exceptions, like *metE* gene at 2 h of starvation (Supplemental Table S3). However, a significant difference was observed in the ratio of the full-length to the truncated transcripts, with an overrepresentation of the truncated transcript in methionine presence and a shift towards full-length transcript under methionine starvation, reflecting an activation of the methionine synthesis. The largest changes in the quantitative composition were observed at 2 h methionine-starvation time point. At 0 h time point (after the transfer to a new media) in turn, we generally observed the lowest total expression level, except for *samT* transcript. Thus, for most of the studied genes, the appearance of the full-length transcripts seems to be caused by a novel transcription under low concentration of methionine, which does not activate the riboswitch-controlled PTT (Fig. 5). This is particularly visible at 2 h time point for *metIC*, *metE*, and *mtnKA* genes/operons and at 3 h time point for *metIC* and *metE* genes. *mtnKA* operon does not show significant changes in induction ratio, comparable to the control — *gyrA*, suggesting that riboswitch domain detected in its 5ʹ UTR could be acting via translation regulation or being inactive pseudo-riboswitch.

**Fig. 6:**
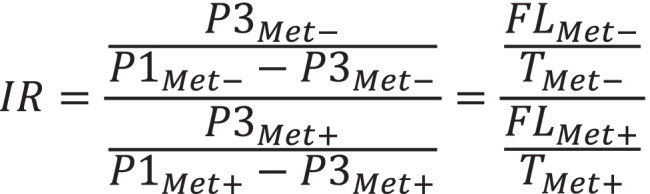
The induction ratio calculation. IR — induction ratio. FL_Met-_ — the concentration of the full-length transcript in methionine starvation conditions. FL_Met+_ — the concentration of the full-length transcript in methionine presence conditions. T_Met-_ — the concentration of the terminated transcript in methionine starvation conditions. T_Met+_ — the concentration of the terminated transcript in methionine presence conditions. P1_Met-_ — the concentration of the product amplified with the primers 1 in methionine starvation conditions. P1_Met+_ — the concentration of the product amplified with the primers 1 in methionine presence conditions. P3_Met-_ — the concentration of the product amplified with the primers 3 in methionine starvation conditions. P3_Met+_ — the concentration of the product amplified with the primers 3 in methionine presence conditions

Based on the full-length and the truncated transcripts concentration estimated with the use of the ddPCR analysis, we calculated the induction ratios of the SAM riboswitches in *B. subtilis*, defined as a ratio between the normalized amount of the full-length transcripts in a holo state of the riboswitch (induced by methionine starvation, Met-) to the apo state (in the presence of methionine, Met +) (Fig. 6). The induction ratio (IR) was calculated separately for four time points.

**Fig. 7:**
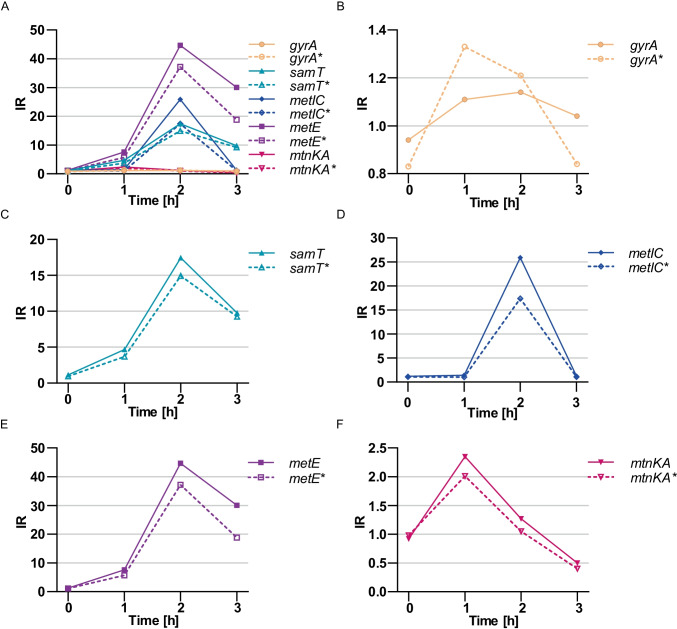
The induction ratio of SAM riboswitches measured by ddPCR. The samples were treated with RNase H cleavage with (solid lines) and without DNA oligomers (dashed lines). The induction ratio (IR) was calculated at four time points (0 h to 3 h) of methionine elimination. The analysis is presented for: combined data (**A**), *gyrA* (**B**), *samT* (**C**), *metIC* (D), *metE* (**E**), and *mtnKA* (**F**)

All analyzed genes/operons, which expression is controlled by the riboswitches showed a gradual increase in the IR, starting from 1 for a 0 h time point, with a peak in 2 h and a final decrease after 3 h of methionine starvation (Fig. 7A). *mtnKA* operon had a slightly different characteristic, it achieved the highest IR 1 h after methionine elimination and a gradual decrease afterwards (Fig. 7F). The expression of *gyrA* does not show significant induction at any time point (Fig. 7B), as expected for a control gene, not regulated by any riboswitch. The highest IR increase was observed for *metE* gene at 2 h time point (IR = 45) (Fig. 7E), followed by *metIC* operon with IR = 26 (Fig. 7D) and *samT* with IR = 17 (Fig. 7C). *mtnKA* operon only to a small extent responded to changes in methionine levels, reaching the highest IR = 2.5 after 1 h of methionine starvation (Fig. 7F). The introduction of the RNase H cleavage to the procedure eliminated the influence of the distance from the 3ʹ end on estimated transcripts concentration. For all genes controlled by the riboswitches, the introduction of DNA oligomer and RNase H cleavage resulted in an increase in IR relative to the control, by an average of 23% (depending on the gene and time point, even up to 60%, Table 1).

**Table 1:** The comparison of the induction ratios between samples with and without RNase H cleavage

Gene	RNase H cleavage	0 h	1 h	2 h	3 h
***gyrA***	**-**	0.83	1.33	1.21	0.84
	** + **	0.94	1.11	1.14	1.04
Difference [%]		**14**	** − 17**	** − 6**	**25**
***samT***	**-**	0.96	3.69	14.93	9.24
	** + **	1.14	4.67	17.44	9.75
Difference [%]		**18**	**26**	**17**	**5**
***metIC***	**-**	1.07	1.02	17.38	1.07
	** + **	1.21	1.43	25.91	1.25
Difference [%]		**13**	**40**	**49**	**17**
***metE***	**-**	1.08	5.72	37.21	18.79
	** + **	1.21	7.60	44.66	30.02
Difference [%]		**11**	**33**	**20**	**60**
***mtnKA***	**-**	0.98	2.01	1.05	0.40
	** + **	0.92	2.35	1.27	0.50
Difference [%]		** − 6**	**17**	**21**	**27**

The difference in the induction ratios is presented in bold

### SAM riboswitch activity with RNaseH/ddPCR is in agreement with qRT-PCR method

Finally, we decided to compare the utility of the newly established protocol by comparison of the transcriptional activity of the riboswitches based on the results obtained using ddPCR method (DD tests) and RT-qPCR method (RT tests). We reasoned that such comparison will allow for the final assessment of the usefulness and the adequacy of the newly developed method in relation to the existing one.

**Fig. 8:**
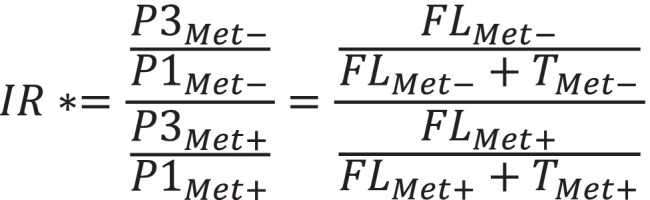
The new induction ratio IR* for comparison of ddPCR and RT-qPCR method. IR* — the new induction ratio. FL_Met-_ — the concentration of the full-length transcript in methionine starvation conditions. FL_Met+_ — the concentration of full-length transcript in methionine presence conditions. T_Met-_ — the concentration of the terminated transcript in methionine starvation conditions. T_Met+_ — the concentration of the terminated transcript in methionine presence conditions. P1_Met-_ — the concentration of the product amplified with the primers 1 in methionine starvation conditions. P1_Met+_ — the concentration of the product amplified with the primers 1 in methionine presence conditions. P3_Met-_ — the concentration of the product amplified with the primers 3 in methionine starvation conditions. P3_Met+_ — the concentration of the product amplified with the primers 3 in methionine presence conditions

We have selected two riboswitches for the comparison: with the highest IR (*metE*) and the lowest IR (*mtnKA*) and performed a quantitative real-time PRC analysis (Supplemental Table S4). Based on the obtained data, we calculated the riboswitch induction ratios (Supplemental Table S5). However, the induction ratio in RT-qPCR method had to be calculated in a different way than in RNaseH/ddPCR method, as FL/TT ratio cannot be calculated from RT-qPCR results (only FL/(FL + TT) ratio). The reason is the fact that PTT products cannot be directly quantified with the use of RT-qPCR. So as to compare both methods, new induction ratio (IR*) was applied as shown below (Fig. 8).

**Fig. 9:**
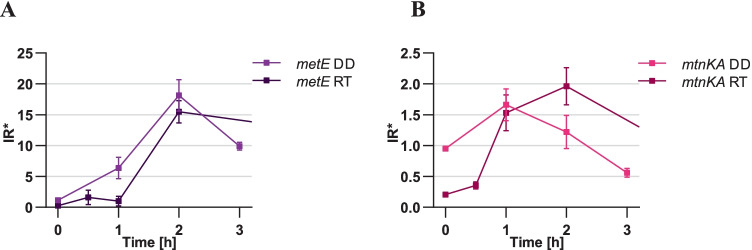
Comparison of the new IR* between ddPCR and qRT-PCR. The new induction ratio* was calculated at four time points (0 h to 3 h) of methionine elimination with the use of two different methods: ddPCR (brighter colors, DD) and qRT-PCR (darker colors, RT). The analysis was performed for two riboswitch-containing genes: *metE* (**A**) and *mtnKA* (**B**)

The dynamic of the IR* changes for *metE* gene was very similar for both techniques, with an increasing IR* up to the highest value of 16 at a methionine starvation time point of 2 h, followed by a gradual decrease (Fig. 9A, Supplemental Table S5). Particular, ddPCR (DD) samples generally reached higher values. In case of *mtnKA* operon, both techniques agreed on the lack of induction of this operon due to the lack of methionine at all time points (Fig. 9B, Supplemental Table S5).

### The connection between SAM riboswitch induction profiles and their metabolic function

A thorough analysis of the expression profiles of different genes/operons led us to several characteristic conclusions. Although different genes/operons are controlled by the same class of SAM-I riboswitch, the expression profile over time is different, which refers to both the strength of the induction and the time point at which this induction occurs. For example, the expression of *samT* and *metE* is significantly induced 1 h after methionine depletion (~ 4.7 and ~ 7.6 fold, respectively, Supplemental Table S5). Both genes encode methionine syntheses (Supplemental Fig. S5), directly involved in amino acid production. The early activation of these proteins during methionine starvation might serve as an adaptive mechanism, which allows bacteria to quickly compensate for this essential amino acid, protecting against dangerous deficiency. On the other hand, the *metIC* operon is engaged in the biosynthesis of important intermediates in the methionine biosynthesis pathway, cysteine, and cystathionine. Due to their indirect role in methionine compensation, the expression is activated at 2 h time point. Among all analyzed genes/operons, the *metE* is characterized by the highest IR in response to methionine concentration (~ 45 fold). It is responsible for biosynthesis of the main and most efficient enzyme producing methionine, which explains the significant increase in its induction. Genes of the *mtnKA* operon respond weakly to the methionine starvation, reaching a peak of IR (~ 2.4 fold) (Supplemental Table S5) at the 1 h time point. These genes are involved in further steps of the methionine salvage pathway and are not critical to bacterial survival. For that reason, their expression does not have to be controlled by SAM concentration as tightly as previous genes/operons.

## Discussion

The data from variety of RNA-seq experiments suffer from different kinds of biases in the context of coverage along a gene’s length. The most influential factor is direct cDNA library preparation. Hence, a relative overrepresentation of 3′ end-derived amplification products with the use of poly(T) adapters (Levin et al. 2010) or the overrepresentation of 5′ end derived amplification products is observed (Agarwal et al. 2015). This problem cannot be neglected as both ends of the gene contain crucial information about regulatory nature of the gene, like transcription termination site, premature termination site, or polyadenylation site. All kinds of biases are not only limited to RNA-seq techniques, but might relate to all molecular methods, as long as cDNA synthesis is involved (e.g. RT-qPCR or ddPCR).

Taking the advantage of unique properties of the RNase H enzyme and the digital droplet PCR technique, we developed a new method for the quantitative analysis of the transcriptional activity of *B. subtilis* SAM riboswitches. The method proposed herein overcomes the barriers in this process like quantification of transcript in an absolute manner or the bias in a number of transcripts depending on the distance from the 3ʹ end. To date, terminated transcripts could not be easily and precisely quantified; thus, the main attention was focused only on full-length transcripts (Tomsic et al. 2008). Owing to the RT-ddPCR technique, we were able for the first time, to present absolute concentrations of both full-length and PTT transcripts. The application of RNase H cleavage at the position defined by the DNA oligomer eliminated the impact of the distance from its 3ʹ end on the number of calculated transcripts.

The in vitro and ex vivo experiments presented herein proved that RNase H is capable of cleaving any RNA molecule efficiently and specifically, even in a pool of total RNA. It is worth noting that mRNA constitutes only ~ 5% of the total RNA of bacterial cells, not to mention the specific transcript of interest (Westermann et al. 2012). For that reason, achieving an acceptable specificity for the reaction was particularly challenging. The use of the RT-ddPCR gave us the opportunity to more accurately determine the cleavage efficiency, reaching almost 100%.

The real challenge for the developed method is to apply it to solve an independent research problem. In this case, an attempt was made to determine the concentration of full-length and PTT transcripts, as well as calculating an improved IR induction ratio of *B. subtilis* SAM riboswitches. In addition, the IR coefficient was determined both for samples subjected to RNase H cleavage (with DNA oligomer) and for control samples (without DNA oligomer), which allowed to trace the impact of the method on the result obtained. It turned out to be significant and the IR value increased by an average of 23% as a result of RNase H digestion. The average IR increase is consistent with the theoretical assumptions. The cleavage of the transcripts reduces overrepresentation of the reads from the 5ʹ end, which leads to a decrease in the number of reads from primers 1 (Fig. 6). In addition, this effect is the stronger, the greater the proportion of full-length transcripts, which means that the numerator of the equation increases more (for Met- samples) than the denominator (Met +). That is why the strongest effect of RNase H cleavage is observed in case of genes with generally highest expression, like *metIC* or *metE*. They are also characterized with the highest induction ratio. During methionine starvation, the in vivo pool of methionine remains constant up to 1 h, with a significant drop after 1.25 h (Tomsic et al. 2008). It explains the observed increased induction ratio 1 h after the methionine was removed. Similar results were obtained when dd-PCR-derived results were compared to qRT-PCR. The changes of the transcription levels in the course of time showed certain regularities, reflecting the physiological functions of individual genes/operons in methionine metabolism. The genes which are directly involved in methionine biosynthesis, like *metE* or *metIC* operon, need to be tightly controlled in response to methionine starvation; hence, the highest induction ratio was observed. The genes engaged in the recycling of the methionine (e.g., *mtnKA*) do not need to be tightly controlled (Murphy et al. 2002), that is why the induction ratio was lower during methionine starvation. *samT* gene encodes an additional methionine synthase, so its expression is characterized by an average induction ratio.

Considering all the benefits of using the proposed method to perform a transcriptional analysis of the riboswitches, a noteworthy idea would be to extend it to all transcriptional riboswitches. However, it is worth remembering that this method does not have to be limited for the analysis of the riboswitches. Applicability of this method can be easily expanded to the analysis of any regulatory elements based on transcription termination mechanisms and also outside the Bacteria domain (Kamieniarz-Gdula and Proudfoot, 2019). The phenomenon of premature transcription termination is also known to be present in human (Dubbury et al. 2018) and orchestes such important processes as tissue-specific gene expression (Lianoglou et al. 2013). Moreover, premature transcription termination is a key regulatory mechanism in human diseases, like leukemia (Lee et al. 2018). More thorough analysis of this process with the application of the PTT-quant method could be particularly beneficial for the understanding such urgent medical issues.

## Supplementary Information

Below is the link to the electronic supplementary material.

Supplementary file1 (PDF 858 kb)

## Data Availability

On request.
