# Supplementary material for: PTT-quant: a new method for direct identification and absolute quantification of premature transcription termination events, following the example of bacterial riboswitches
Source: Appl Microbiol Biotechnol. 2022 Feb 5;106(4):1557–70. doi: 10.1007/s00253-022-11809-1 (PMC8882085; doi:10.1007/s00253-022-11809-1)
Supplement: Supplementary file 1 — Supplementary file1 (PDF 858 kb) [file 253_2022_11809_MOESM1_ESM.pdf]

Applied Microbiology and Biotechnology

PTT-quant - a new method for direct identification and absolute quantification of premature transcription termination events, following the example of bacterial riboswitches

Piotr Machtel, Anna Wasilewska-Burczyk, Julian Zacharjasz, Kamilla Grzywacz*

Institute of Bioorganic Chemistry Polish Academy of Sciences, Poznań, Poland
*corresponding author:
Email: kamilla.grzywacz@ibch.poznan.pl
Phone: +48/618291864
Fax: +48/618528503

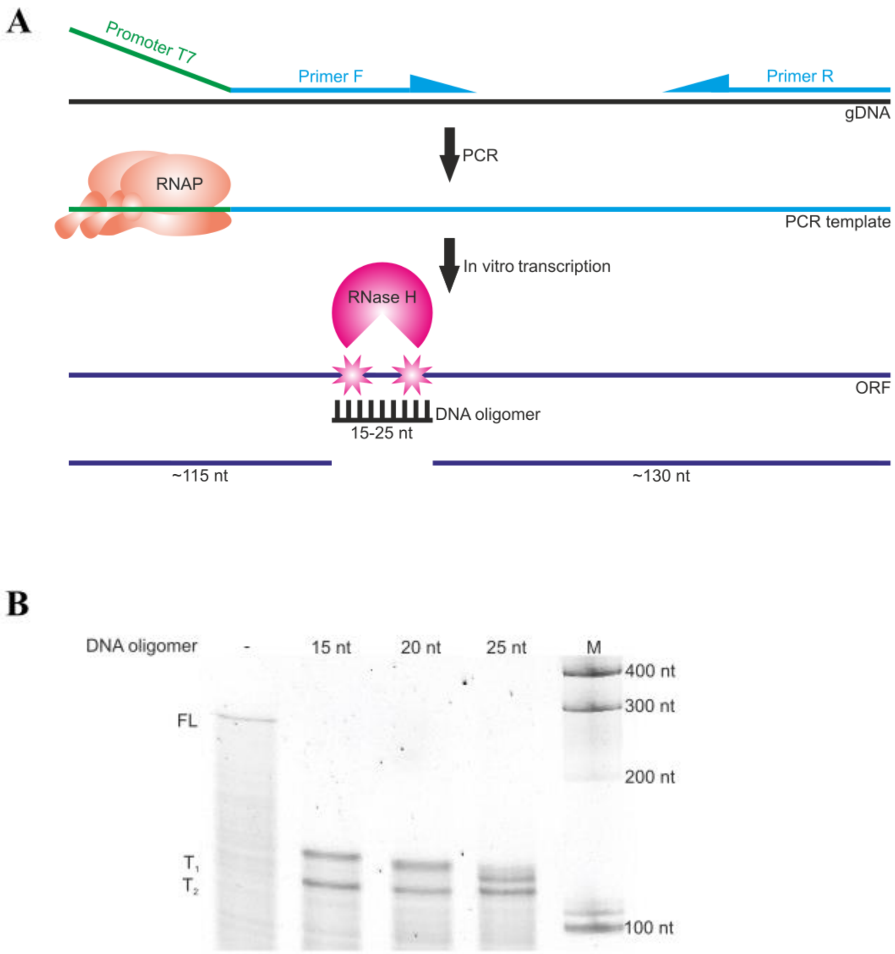

Figure S1. RNase H cleavage of *gyrA* in vitro transcripts with different DNA oligomers: 15 nt, 20 nt and 25 nt long.

Schematic representation of RNase H nucleolytic activity for in vitro transcripts (A) and the experimental results of RNase H activity (B).

M – RNA size marker, FL – full-length transcript, T_1 and T_2 – products of RNase H cleavage. „-" – control sample without DNA oligomer.

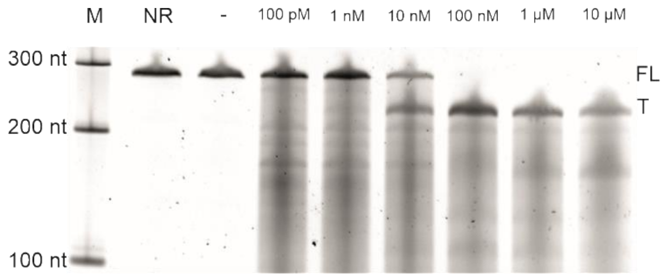

Figure S2. RNase H cleavage of *metE* in vitro transcripts in a growing gradient of a DNA oligomer.
M – RNA size marker, NR - no reaction, "-" – control reaction with no DNA oligomer, FL – full-length transcript, T - RNase H cleavage product.

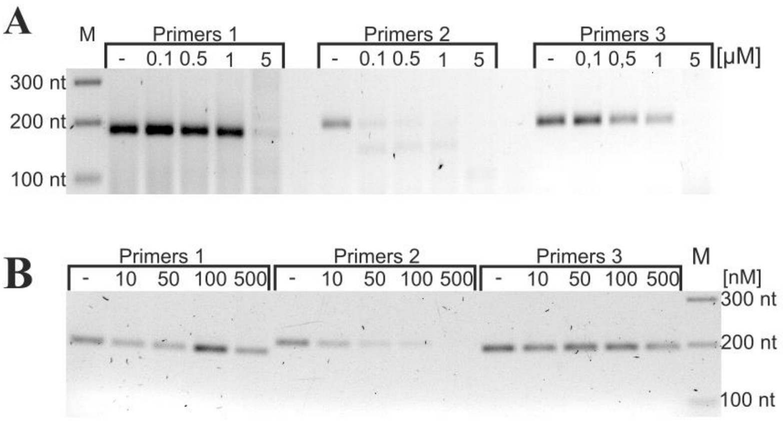

Figure S3. *gyrA* transcripts are efficiently cleaved by RNase H ex vivo in a broad range of DNA oligomer concentrations.
RNase H cleavage of *gyrA* transcripts in the pool of *ex vivo* transcripts was performed in a gradient of DNA oligomer concentrations: 0.1-5 μM (A) and 10-500 nM (B). A clear disappearance of the signals from products amplified solely by primers 2 (F2/R2) is observed, which indicates specific RNase H cleavage. The signals derived from the PCR products of primers 1 and 3 are not significantly decreased.
M - DNA size marker.

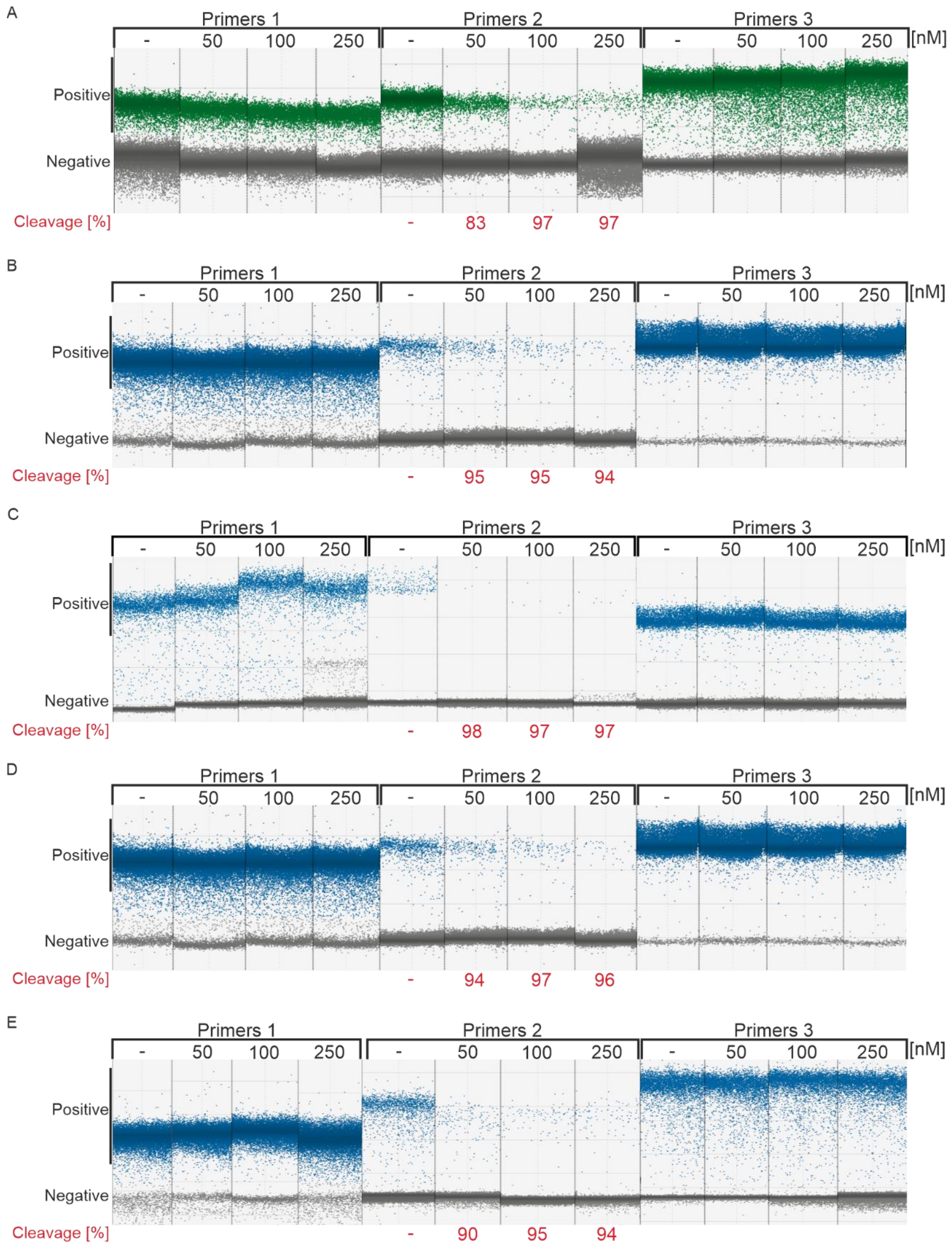

Figure S4. ddPCR result of ex vivo SAM riboswitch-containing transcript after RNase H cleavage: *gyrA* (A), *samT* (B), *metE* (C), *metIC* (D) and *mtnKA* (E).

For each set of primers (primers 1, 2 and 3), a DNA oligomer gradient from 0 nM (-) to 250 nM was applied. Positive droplets are marked as green dots, negative - as gray dots. Positive droplets, due to the presence of the EvaGreen fluorescent dye, emit light only with PCR reaction product inside. The higher input concentration of a given transcript, the more copies of the cDNA and the greater the number of positive versus negative droplets.

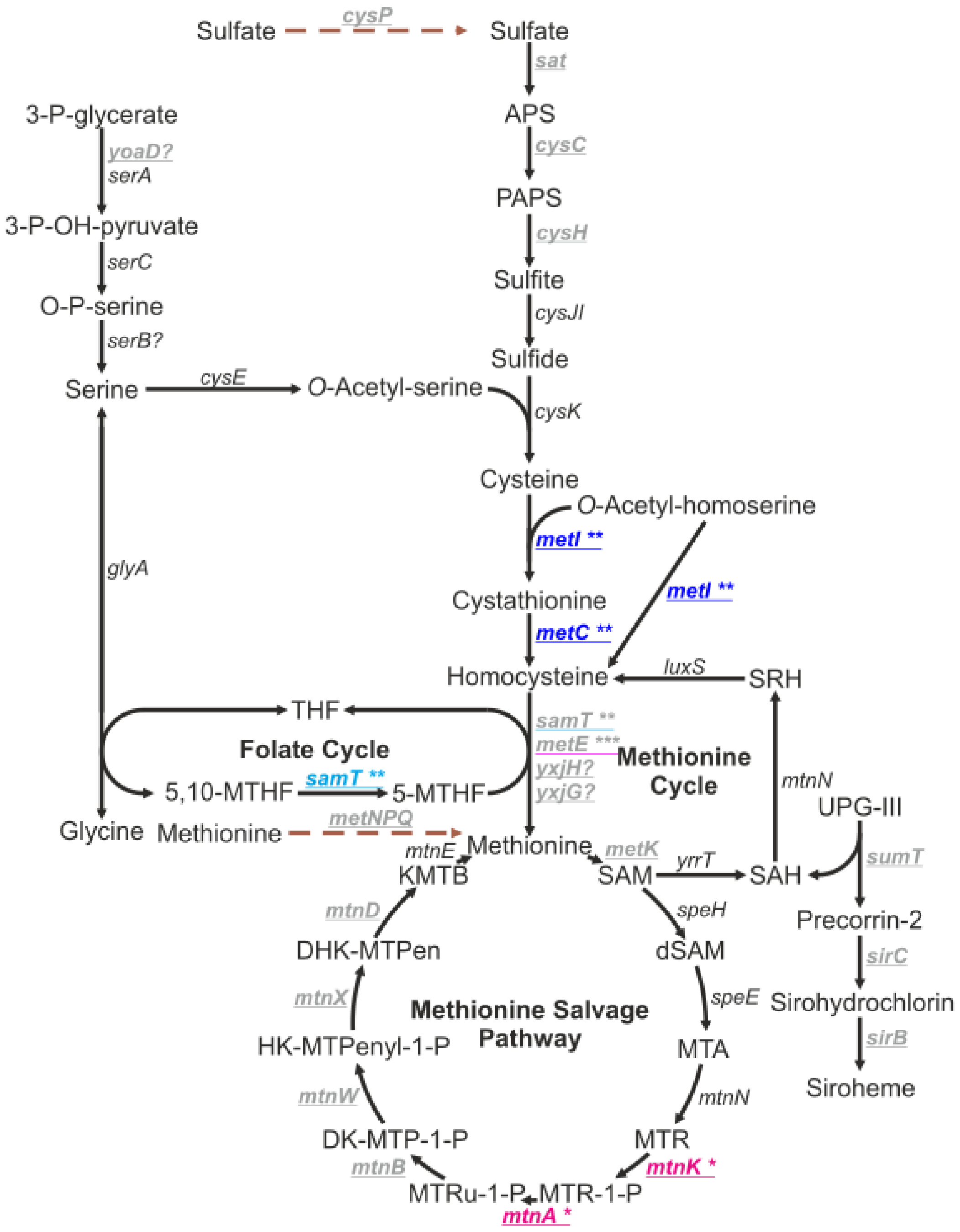

Figure S5. Methionine biosynthesis and metabolism pathway.
Underlined and bold and genes are controlled by SAM-I riboswitch. Browned, dashed arrows – methionine import. APS – adenosine-5′-phosphosulfate. PAPS – 3'-phosphoadenosine-5'-phosphosulfate. THF – tetrahydrofolate. MTHF – methyl-THF. SAM – *S*-adenosylmethionine. dSAM – decarboxylated SAM. SAH – *S*-adenosylhomocysteine. SRH – *S*-ribulosehomocysteine. MTA – 5′-methyladenosine. MTR – 5′-methylthioribose. MTR-1-*P* – MTR-1-phosphate. MTRu-1-*P* – 5-methylthioribulose-1-phosphate. DK-MTP-1-*P* – 2.3-diketo-5-methylthiopentan-1-phosphate. HK-MTPentyl-1-*P* – 2-hydroksy-3-keto-5-methylthiopentenyl-1-phosphate. DHK-MTPen-1-P – 1.2-dihydroksy-3-keto-5-methylthiopenten-1-phosphate. KMTB – 2-keto-4-methylthiobutyrate. UPG-III – uroporphyrinogen-III. Asterisks corresponds with induction ratio: *** – high induction, ** – average induction, * – weak induction.

Table S1. Primers used for PCR, ddPCR and RT-qPCR reaction.

Gene	Primer name	5′-3′ sequence	Length
gyrA	gyrA F1	ATGAGTGAACAAAACACACCAC	22 nt
	gyrA R1	GCTTGTCACTTGTCATGCCT	20 nt
	gyrA F2	GCATGACAAGTGACAAGCCT	20 nt
	gyrA R2	TCTGTATAACGCATGGCCGC	20 nt
	gyrA F3	CCGAGAACAACGGCCAACTA	20 nt
	gyrA R3	GCAACATGCTCTTCTTCTGCC	21 nt
samT	samT F1	ACACGAAAATTTCATATCCGTTCT	24 nt
	samT R1	TGTCTCTTCTTATCTTCCAAGCTGT	25 nt
	samT F2	AAGCTCGAACAGCTTGGAAGA	21 nt
	samT R2	TAGGAGTAGAGGAGCGTCCC	20 nt
	samT F3	GCCCATGCCGGTGAAGATAA	20 nt
	samT R3	ATGATGTGAGCTCAGCGGTT	20 nt
metIC	metIC F1	CGATATTTCTTATCGTGAGAGGTGG	25 nt
	metIC R1	CGCTTCTTCTTATCTTCCAAGCA	23 nt
	metIC F2	TCAGCAACCGGCTTGTTTTG	20 nt
	metIC R2	CTTCATCGCTACGGTTCCCA	20 nt
	metIC F3	GGATATTCCTGAAGAGATCCGCA	23 nt
	metIC R3	GCTCCCTCTTTGACCTGACA	20 nt
metE	metE F1	CTCTTATCGAGAGTTGGGCGAG	22 nt
	metE R1	ACGTAAAACACTGCCTCTCTCA	22 nt
	metE F2	AGGGATTGGCCTTTTGACCC	20 nt
	metE R2	AAACACTCTCTTTCACCCGC	20 nt
	metE F3	AATCGGACTGAACCGGGAAT	20 nt
	metE R3	TCAACTGGATCCCGAAACGG	20 nt
mtnKA	mtnKA F1	TTATCGAGAGTTGGGCGAGG	20 nt
	mtnKA R1	ACATCTATGTCCGCCTCTCTT	21 nt
	mtnKA F2	TTCCATTGTGAAATGGGGCG	20 nt
	mtnKA R2	CCACAGCGGAGCTTTCATTT	20 nt
	mtnKA F3	AGAAGCAATCGGATTTGCGG	20 nt
	mtnKA R3	GTTTTGAATTCCGAACGTTTTTCG	24 nt

Table S2. DNA oligomers used for RNase H cleavage reaction.

Gene	Oligomer name	5′-3′ sequence	Lenght
gyrA	gyrA oligomer 15	GCTGAATCACCGTGC	15 nt
	gyrA oligomer 20	ATACCGCTGAATCACCGTGC	20 nt
	gyrA oligomer 25	TTCATATACCGCTGAATCACCGTGC	25 nt
samT	samT oligomer	GCCTCCTTTATTCACATCAGCAAG	24 nt
metIC	metIC oligomer	TTATTTTGAAAAGGGAAGGT	20 nt
metE	metE oligomer	GGCTGCTAAGAGGTTTTCTCAC	22 nt
mtnKA	mtnKA oligomer	TTATGTAATTAATTAATATGTGGCCG	26 nt

Table S3. The absolute concentrations of the riboswitch-containing transcripts at different time points of methionine starvation.

The concentration expressed in copies of RNA per µl [c/µl] was calculated for the prematurely terminated (T) and full-length (FL) transcripts. PTT ratio was calculated as the ratio of the concentrations between the full-length and prematurely terminated transcripts, according to the formula on Fig.4. DNA oligomer + (A) and DNA oligomer – (B) conditions are presented.

A

Gene	Met	0 h				1 h				2 h				3 h			
		FL+T	T	FL	PTT ratio	FL+T	T	FL	PTT ratio	FL+T	T	FL	PTT ratio	FL+T	T	FL	PTT ratio
samT	–	6 120	6 075	45	0.0074	7 480	6 653	827	0.1243	1 531	550	981	1.7836	1 998	966	1 032	1.0683
	+	5 490	5 454	36	0.0066	5 360	5 221	139	0.0266	2 102	1 907	195	0.1023	1 610	1 451	159	0.1096
metIC	–	4 940	4 659	281	0.0603	31 350	28 595	2 755	0.0963	8 490	3 010	5 480	1.8206	14 190	12 584	1 606	0.1276
	+	4 270	4 067	203	0.0499	16 190	15 171	1 019	0.0672	9 550	8 923	627	0.0703	10 140	9 201	939	0.1021
metE	–	5 870	5 686	184	0.0324	25 770	18 920	6 850	0.3621	32 390	11 360	21 030	1.8512	12 220	3 560	8 660	2.4326
	+	6 330	6 165	165	0.0268	23 750	22 670	1 080	0.0476	13 140	12 617	523	0.0415	14 140	13 080	1 060	0.0810
mtnKA	–	2 780	670	2 110	3.1493	15 700	5 550	10 150	1.8288	7 390	4 640	2 750	0.5927	15 840	12 490	3 350	0.2682
	+	2 700	610	2 090	3.4262	17 240	9 690	7 550	0.7792	10 740	7 320	3 420	0.4672	5 530	3 610	1 920	0.5319

B

Gene	Met	0 h				1 h				2 h				3 h			
		FL+T	T	FL	PTT ratio	FL+T	T	FL	PTT ratio	FL+T	T	FL	PTT ratio	FL+T	T	FL	PTT ratio
samT	–	6 230	6 196	33.8	0.0055	9 830	9 014	816	0.0905	1 709	734	975	1.3283	2 387	1 268	1119	0.8825
	+	6 700	6 662	37.7	0.0057	6 100	5 954	146	0.0245	1 983	1 821	162	0.0890	1 790	1 634	156	0.0955
metIC	–	4 600	4 385	215	0.0490	35 590	33 159	2431	0.0733	8 190	3 600	4 590	1.2750	11 500	10 253	1247	0.1216
	+	4 190	4 006	184	0.0459	15 040	14 036	1004	0.0715	9 950	9 270	680	0.0734	9 890	8 877	1013	0.1141
metE	–	8 320	8 133	187	0.0230	29 550	23 410	6140	0.2623	33 640	15 450	18 190	1.1773	15 270	6 720	8550	1.2723
	+	7 660	7 501	159	0.0212	28 750	27 490	1260	0.0458	16 890	16 372	518	0.0316	17 190	16 100	1090	0.0677
mtnKA	–	2 840	810	2030	2.5062	18 750	8 320	10430	1.2536	8 440	5 420	3 020	0.5572	18 190	14 340	3850	0.2685
	+	2 960	830	2130	2.5663	20 110	12 380	7730	0.6244	11 840	7 740	4 100	0.5297	5 300	3 160	2140	0.6772

Table S4. The C_t values of the riboswitch-containing transcripts at different time points of methionine starvation.

The C_t values was calculated for the prematurely terminated (T) and full-length (FL) transcripts and presented as ± standard deviation (SD) from three independent experiments.

Gene	Met	0 h		0.5 h		1 h		2 h		4 h	
		FL+T [C_t]±SD	FL [C_t]±SD	FL+T [C_t]±SD	FL [C_t]±SD	FL+T [C_t]±SD	FL [C_t]±SD	FL+T [C_t]±SD	FL [C_t]±SD	FL+T [C_t]±SD	FL [C_t]±SD
metE	–	28.54 ±0.12	33.25 ±0.48	24.51 ±0.25	31.59 ±0.03	23.21 ±0.23	34.43 ±0.02	23.91 ±0.63	31.19 ±0.17	22.03 ±0.09	31.8 ±0.23
	+	29.83 ±0.16	32.63 ±0.07	26.9 ±0.19	34.87 ±0.54	24.8 ±0.02	38.38 ±0.11	26.12 ±0.09	33.43 ±0.02	18.86 ±0.12	32.39 ±0.69
mtnKA	–	28.53 ±0.02	33.35 ±0.14	24.27 ±0.15	32.13 ±0.06	20.74 ±0.04	34.17 ±0.2	25.55 ±0.28	32.88 ±0.11	25.87 ±0.18	32.02 ±0.16
	+	30.27 ±0.1	32.78 ±0.07	27.73 ±0.27	34.19 ±0.32	20.95 ±0.01	35.15 ±0.1	25.93 ±0.22	33.88 ±0.16	26.9 ±0.06	32.84 ±0.04

Table S5. IR* results achieved by ddPCR and RT-qPCR for *metE* and *mtnKA*.

**Gene**	**Method**	**0 h**	**1 h**	**2 h**	**3 h**
		**IR*±SD**	**IR*±SD**	**IR*±SD**	**IR*±SD**
metE	ddPCR	1.13±0.11	6.38±0.75	18.12±2.55	9.90±0.63
mtnKA	ddPCR	0.95±0.04	1.91±0.62	1.22±0.07	0.56±0.07
metE	RT-qPCR	0.27±0.12	1.00±0.79	15.50±1.80	12.80±4.60
mtnKA	RT-qPCR	0.21±0.04	1.53±0.29	1.96±0.30	0.87±0.27
